# Outcome of post-traumatic acute respiratory distress syndrome in young patients requiring extracorporeal membrane oxygenation (ECMO)

**DOI:** 10.1038/s41598-022-14741-6

**Published:** 2022-06-23

**Authors:** Hassan Al-Thani, Ammar Al-Hassani, Ayman El-Menyar, Mohammad Asim, Ibrahim Fawzy

**Affiliations:** 1 Department of Surgery, Trauma Surgery, Hamad General Hospital, Doha, Qatar; 2 Clinical Research, Trauma & Vascular Surgery, Department of Surgery, Hamad General Hospital, Doha, Qatar; 3 Department of Clinical Medicine, Weill Cornell Medical School, Doha, Qatar; 4 Department of Internal Medicine, Hamad General Hospital, Doha, Qatar

**Keywords:** Diseases, Health care, Medical research, Nephrology

## Abstract

We aimed to evaluate the outcomes of post-traumatic acute respiratory distress syndrome (ARDS) in young patients with and without Extracorporeal membrane oxygenation (ECMO) support. A retrospective analysis was conducted for trauma patients who developed ARDS at a level I trauma facility between 2014 and 2020. Data were analyzed and compared between ECMO and non-ECMO group. We identified 85 patients with ARDS (22 patients had ECMO support and 63 matched patients managed by the conventional mechanical ventilation; 1:3 matching ratio). The two groups were comparable for age, sex, injury severity score, abbreviated injury score, shock index, SOFA score, and head injury. Kaplan Meier survival analysis showed that the survival in the ECMO group was initially close to that of the non-ECMO, however, during follow-up, the survival rate was better in the ECMO group, but did not reach statistical significance (Log-rank, p = 0.43 and Tarone-Ware, p = 0.37). Multivariable logistic regression analysis showed that acute kidney injury (AKI) (Odds ratio 13.03; 95% CI 3.17–53.54) and brain edema (Odds ratio 4.80; 95% CI 1.10–21.03) were independent predictors of mortality. Sub-analysis showed that in patients with severe Murray Lung Injury (MLI) scores, non-ECMO group had higher mortality than the ECMO group (100% vs 36.8%, p = 0.004). Although ARDS is uncommon in young trauma patients, it has a high mortality. ECMO therapy was used in a quarter of ARDS cases. AKI and brain edema were the predictors of mortality among ARDS patients. ECMO use did not worsen the outcome in trauma patients; however, the survival was better in those who had severe MLI and ECMO support. Further prospective study is needed to define the appropriate selection criteria for the use of ECMO to optimize the outcomes in trauma patients.

## Introduction

Trauma is the frequent cause of mortality in severely injured young patients secondary to hemorrhagic shock and cardiopulmonary dysfunction. Therefore, controlling active bleeding and maintaining arterial oxygenation are essential to improve the patients’ outcome^1,2^. Around 10–20% of polytrauma patients may develop severe respiratory complications which necessitate ventilatory support^3,4^. Among such patients, acute respiratory distress syndrome (ARDS) remains a challenging complication which may occur in 6.5% of patients requiring mechanical ventilation for greater than 48 h^5^. Notably, ARDS is multifactorial that could be related to direct thoracic trauma and/or indirect injury caused by extrapulmonary trauma and its management such as massive transfusion, fluid overload, and ventilator-induced acute lung injury^6^.

Despite advances in pulmonary critical care management with adoption of lung protective ventilation, the mortality rate remains high among trauma patients with ARDS (17–46%)^7–9^. For instance, it is challenging to apply adjunctive measures such as prone positioning with conventional ventilator management strategies for ARDS in patients with brain, spine, or pelvic injuries. Moreover, permissive hypercapnia may be difficult to effectively manage ARDS in patients with traumatic brain injury^9,10^.

Extracorporeal membrane oxygenation (ECMO) has been used as a salvage therapy in patients with unsuccessful or unsatisfactory conventional ventilatory support. It is effective in treating hypoxic respiratory failure caused by traumatic lung injury. This may be related to the benefit of warming, correction of acidosis, better oxygenation, and circulatory support^11^. Some studies have reported survival rates between 50–79% after the utilization of ECMO in trauma^12–15^. Although, the use of ECMO in non-trauma setting continues to expand, its utility in trauma patients remains controversial or inaccessible in many centers. Limited resources, bleeding, thrombosis, limb ischemia, traumatic brain injury, complicated pelvic fractures, major vascular injuries, and lack of technical expertise are the major factors affecting the widespread use of ECMO in trauma patients^16^. The use of ECMO in trauma patients is recently adopted in selected cases in our trauma center at Hamad Medical Corporation (HMC). To date, data on the use of ECMO in trauma patients are underreported in our region in the Arab Middle East. The present study aims to evaluate the outcomes of post-traumatic ARDS in young patients with and without the use of ECMO in a level-1 trauma center and to look for the role of acute kidney injury (AKI) in those patients as well. We hypothesized that the use of ECMO in trauma patients with ARDS is associated with better outcomes.

## Methods

A retrospective analysis of trauma patients who developed and treated for severe ARDS at the level I trauma facility at Hamad Medical Corporation between January 2014 and January 2020 was conducted. Medical records of adult patients of all genders with severe ARDS were reviewed. All ARDS patients were diagnosed based on the Berlin definition of hypoxemia PaO2/FIO2 < 200, FiO2 0.8–1.0, positive end-expiratory pressure ≥ 5 cm H_2_O and bilateral pulmonary infiltrates that are not entirely caused by cardiac failure^17^. Inquired data from the trauma registry included patient demographics, mechanism of injury (MOI), diagnoses, hemodynamic parameters, Glasgow coma scale (GCS), injury severity score (ISS), abbreviated injury scale (AIS) and associated injuries. Type of cannulation [veno-venous (VV) or veno-arterial (VA)], time from admission to ECMO, exploratory laparotomy, tracheostomy, thoracotomy, open reduction and internal fixation (ORIF) surgery, external fixation, intracranial pressure (ICP) monitoring, craniectomy, angioembolization, ventilatory days, ICU and hospital length of stay, complications and outcomes were recorded. Complications included sepsis, limb ischemia, disseminated intravascular coagulation (DIC), deep vein thrombosis (DVT), pulmonary embolism (PE), AKI, cerebrovascular accident (CVA), multiorgan failure (MOF), ventilator associated pneumonia (VAP), thrombocytopenia, and bleeding. Traumatic injuries were defined according to ICD-9 codes.

The primary outcome measure of the study was 30-day post-admission and > 30-day follow-up mortality.

Secondary outcome measures included AKI in addition to hospital length of stay (LOS), ICU-LOS, ventilator days and multiorgan failure (MOF).

AKI was defined according to the Kidney Disease Improving Global Outcomes (KDIGO) criteria^18^. MOF was defined as physiological abnormalities with dysfunction (reversible or irreversible) of two or more organs (i.e., lung, kidney, liver, coagulation, central nervous system (CNS) and heart) that occurs simultaneously leading to longer stay in the intensive care unit and high risk of mortality^19,20^.

### Scoring systems

Shock index (SI), abbreviated injury score (AIS), Injury severity core (ISS), and Sequential Organ Failure Assessment (SOFA) score. SI was defined as admission heart rate divided by systolic blood pressure (normal value 0.5–0.7)^21,22^. The respiratory ECMO survival prediction (RESP) scores were calculated for each patient, which ranges between − 22 and 15. The survival rate varies according to RESP score from 92% in RESP class I, 76% class II, 57% class III, 33% class IV and 18% class V^23^. Also, Murray Lung Injury (MLI) scores were determined at the time of severe ARDS diagnosis; a MLI score of 3.0 or greater suggests that the patient is hypoxic and may benefit from ECMO support^24^.

### Pulmonary and ECMO management

ARDSNet protocol goals were used as a general guideline for oxygenation, ventilation, pH, and airway pressure management. Initial modes of ventilation included both volume control and pressure control modes to maintain lung-protective ventilation (tidal volume ≤ 6 mL/kg per ideal body weight). Additional adjuncts were initiated as deemed appropriate by the trauma and ECMO intensivist before the initiation of ECMO. The adjuncts included muscle relaxant, nitric oxide, and/or reverse I/E ventilation and prone positioning. Patients who were selected for ECMO therapy were those with severe ARDS and persistent hypoxia despite maximal mechanical ventilator support with or without adjuncts. All patients treated with ECMO for refractory hypoxia were transferred from the trauma intensive care unit to the medical intensive care unit. In case of traumatic brain injury (TBI), no heparin was given for 48–72 h post-trauma.

### Ethics approval and consent to participate

This observational study has received expedited review and was approved by the Institutional Review Board, Medical Research Center (MRC-01-20-503) at Hamad medical corporation (HMC), Doha, Qatar which waived the need of informed consent due to retrospective nature of the study. All methods were performed in accordance with the relevant guidelines and regulations. Informed consent was not required as data were retrospectively and anonymously collected and kept confidential without direct contact with the patients**.**

### Statistical analysis

Data were expressed as numbers, percentages, mean ± standard deviation or medians with range, whenever appropriate. Chi-square test was performed for the analysis of differences in categorical variables between ECMO vs. non-ECMO groups, and Fisher exact test was used when the observed cell values were < 5. The continuous variables between different groups were compared using student’s t test and the two-tailed p values < 0.05 were considered as significant. The ECMO group was matched with the non-ECMO group (1:3 matching ratio) in terms of age, sex, ISS and AIS. Multivariable logistic regression analysis was performed for predictors of mortality among trauma patients with ARDS using the following variables: age, ISS, GCS, SI, Murray score, ECMO use, sepsis, brain edema, extra-axial hematoma, and AKI. Data were expressed as odds ratio and 95% confidence interval (CI). Kaplan–Meier survival curve was used to analyze ‘time-to-event’ data. The outcome (event) was all-cause mortality. Data analysis was carried out using the Statistical Package for Social Sciences version 26 (SPSS Inc. Chicago, Illinois, USA).

## Results

### Overall study population

During the study period, a total of 85 patients with severe ARDS (22 patients were treated with ECMO and 63 matched patients were managed without ECMO) were included in the study (1:3 matching ratio). Seventy-eight (91.8%) patients were males and the mean age of the cohort was 34.3 ± 14.9 years. The mean SI and ISS were 1.05 ± 0.47 and 30.4 ± 13.6, respectively (Table 1). Thoracic injury accounted for the highest proportion of trauma diagnoses (76.5%) followed by TBI (55.3%) and abdominal injury (51.8%). Overall, the most frequent complications were AKI (38.8%) and VAP (38.8%), followed by sepsis (35.5%) and MOF (16.5%) (Table 1).

**Table 1:** Demographics, clinical characteristics of trauma patients with acute respiratory distress disorder.

Variables	Overall (n = 85)	No-ECMO (n = 63)	ECMO (n = 22)	P
Age (mean ± SD)	34.3 ± 14.9	35.9 ± 15.1	29.6 ± 13.8	0.09
Males	78 (91.8%)	59 (93.7%)	19 (86.4%)	0.28
**Mechanism of injury**				
Motor vehicle crash	38 (44.7%)	27 (42.9%)	11 (50.0%)	0.49 for all
Pedestrian	20 (23.5%)	13 (20.6%)	7 (31.8%)	
Fall from height	16 (18.8%)	13 (20.6%)	3 (13.6%)	
Struck by a heavy Object	3 (3.5%)	2 (3.2%)	1 (4.5%)	
Assault	4 (4.7%)	4 (6.3%)	0 (0.0%)	
Others	4 (4.7%)	4 (6.3%)	0 (0.0%)	
**Initial vitals in ED, severity and scoring tools**				
Systolic blood pressure	109.6 ± 38.3	109.3 ± 40.6	110.3 ± 31.4	0.91
Diastolic blood pressure	66.5 ± 26.3	65.5 ± 26.6	69.5 ± 25.9	0.54
Pulse rate	108.9 ± 24.4	104.3 ± 22.7	121.6 ± 24.9	0.004
Respiratory rate	22.3 ± 4.9	21.8 ± 4.7	23.4 ± 5.5	0.20
Oxygen saturation	92.4 ± 14.2	93.6 ± 14.7	88.9 ± 12.6	0.18
Glasgow coma scale	12 (3–15)	13 (3–15)	9.5 (3–15)	0.18
Shock Index	1.05 ± 0.47	1.00 ± 0.46	1.19 ± 0.47	0.11
SOFA score	9.3 ± 3.4	9.3 ± 2.8	9.4 ± 4.8	0.91
Murray lung injury score	2.40 ± 0.76	2.06 ± 0.47	3.35 ± 0.62	0.001
Severity of lung injury*				0.001 for all
Severe lung injury	26 (30.6%)	7 (11.1%)	19 (86.4%)	
Mild-to-moderate lung injury	59 (69.4%)	56 (88.9%)	3 (13.6%)	
**Injured region**				
Head injury	47 (55.3%)	32 (50.8%)	15 (68.2%)	0.15
Chest injury	65 (76.5%)	47 (74.6%)	18 (81.8%)	0.49
Abdomen injury	44 (51.8%)	31 (49.2%)	13 (59.1%)	0.42
Pelvis injury	26 (30.6%)	17 (27.0%)	9 (40.9%)	0.22
**Complications**				
Acute Kidney Injury	33 (38.8%)	20 (31.7%)	13 (59.1%)	0.02
Ventilator-associated pneumonia	33 (38.8%)	24 (38.1%)	9 (40.9%)	0.81
Sepsis	30 (35.3%)	19 (30.2%)	11 (50.0%)	0.09
Multiorgan failure	14 (16.5%)	7 (11.1%)	7 (31.8%)	0.02
Bowel Ischemia	4 (4.7%)	2 (3.2%)	2 (9.1%)	0.25
AKI required dialysis	10 (11.8%)	3 (4.8%)	7 (31.8%)	0.002
Disseminated intravascular coagulation	4 (4.7%)	2 (3.2%)	2 (9.1%)	0.25
Pulmonary embolism	5 (5.9%)	3 (4.8%)	2 (9.1%)	0.45
Deep vein thrombosis	5 (5.9%)	1 (1.6%)	4 (18.2%)	0.02
Cerebrovascular accidents	4 (4.7%)	3 (4.8%)	1 (4.5%)	0.96
Thrombocytopenia	5 (5.9%)	2 (3.2%)	3 (13.6%)	0.07
Gastrointestinal bleeding	3 (3.5%)	1 (1.6%)	2 (9.1%)	0.10

*Murray lung injury score.

Thromboembolic complications were recorded in 14 patients (5 pulmonary embolism, 5 DVT and 4 CVA). The overall mortality was 41.2% (57% within 30-day post-admission and 43% during follow-up period).

### ECMO group

**Figure 1:**
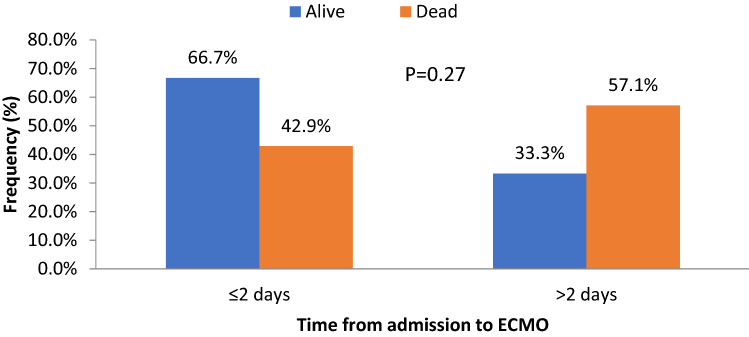
Outcome by time from admission to ECMO.

VV ECMO was the main technique in the ECMO group except one, who received VA ECMO (Table 2). Median time between admission and commencing ECMO was 2 (1.0–14) days. The average time spent on ECMO was 9.5 (1–29) days. Nine patients (40.9%) had EMCO started after 48 h of admission (delayed) and 13 patients (59.1%) had ECMO started within the first 48 h of admission (early) (Table 2 and Fig. 1). Almost two thirds of early ECMO patients had survived compared to one third of patients with delayed ECMO, but this difference did not reach statistical significance (p = 0.27). Peripheral ischemia was noted in 2 cases (1 hand ischemia secondary to arterial line and 1 with foot gangrene secondary to inotrope and delayed distal perfusion). The distribution of patients according to the criteria of RESP scoring is shown in Table 2. Almost 82% of cases (n = 18) were classified as RESP risk class IV-V, of them 6 (33%) died (3 TBI, 2 MOF and 1 sepsis).

**Table 2:** Scores, type and time of ECMO and survival (n = 22).

Variables	Values
**Left ventricular ejection fraction %**	48.0 ± 12.0
**RESP score (mean ± SD)**	4.5 ± 2.5
(median, range)	4.5 (0–11)
**Days on ECMO**	9.5 (1–29)
**Risk class according to RESP score**	**Number of cases**
Risk Class II^a^	1 (4.5%)
Risk Class III^b^	3 (13.6%)
Risk Class IV^c^	13 (59.1%)
Risk Class V^d^	5 (22.7%)
**ECMO type**	**Number of cases**
Veno-venous	21 (95.5%)
Veno-arterial	1 (4.5%)
**Admission to ECMO (median, range) days**	2 (1.0–14)
≤ 2 days	13 cases (59.1%)
> 2 days	9 cases (40.9%)

^a^1 died with cardiac arrest 4-month post-discharge.

^b^1 died with MOF at 30-day.

^c^1 died with sepsis at 30-day and 2 died with MOF after 2 months.

^d^3 died with TBI (1 at 26 days, 1 at 35 days and 1 at 54 days).

### ECMO versus non-ECMO

There was no statistically significant difference between the two groups regarding age, MOI, SI, ISS, SOFA score, TBI, and AIS for head, chest, abdomen and pelvis (Tables 1 and 3). With regards to clinical parameters, there was no difference between the initial vitals (SBP, DBP and Oxygen Saturation) and GCS between the two groups, whereas the initial pulse rate was significantly higher in the ECMO group, (p = 0.004). TBI lesions were comparable in the 2 groups apart from brain edema and extra-axial hematoma that were higher in the ECMO group (p = 0.004) (Table 3). Two patients had bronchus tear in ECMO group and 2 had aortic injury in the non-ECMO group.

**Table 3:** Injury characteristics, types of interventions, outcomes and cause of death.

Variables	Overall (n = 85)		No-ECMO (n = 63)		ECMO (n = 22)		P
Brain Contusion	33 (38.8%)		21 (33.3%)		12 (54.5%)		0.07
Brain Edema	18 (21.2%)		10 (15.9%)		8 (36.4%)		0.04
Subdural hematoma (SDH)	13 (15.3%)		10 (15.9%)		3 (13.6%)		0.80
Epidural hematoma (EDH)	10 (11.8%)		9 (14.3%)		1 (4.5%)		0.22
Extra-axial hematoma	9 (10.6%)		4 (6.3%)		5 (22.7%)		0.03
Subarachnoid hemorrhage (SAH)	20 (23.5%)		16 (25.4%)		4 (18.2%)		0.49
Head AIS	3.9 ± 0.9		4.0 ± 0.9		3.9 ± 0.9		0.81
Chest AIS	3.1 ± 0.7		3.1 ± 0.8		3.0 ± 0.6		0.75
Abdomen AIS	3.0 ± 1.1		2.9 ± 1.2		3.1 ± 0.9		0.76
Pelvis AIS	2.8 ± 1.1		2.7 ± 1.1		3.0 ± 1.1		0.43
Injury Severity Score	30.4 ± 13.6		30.3 ± 14.1		30.6 ± 12.3		0.93
Exploratory laparotomy	34 (40.0%)		23 (36.5%)		11 (50.0%)		0.26
Tracheostomy	36 (42.4%)		22 (34.9%)		14 (63.6%)		0.01
Thoracotomy	6 (7.1%)		4 (6.3%)		2 (9.1%)		0.66
ORIF surgery	23 (27.1%)		16 (25.4%)		7 (31.8%)		0.55
External fixation	12 (14.1%)		7 (11.1%)		5 (22.7%)		0.17
ICP monitoring	19 (22.4%)		7 (11.1%)		12 (54.5%)		0.001
Craniectomy	4 (6.1%)		2 (4.5%)		2 (9.1%)		0.46
Angioembolization	10 (11.8%)		6 (9.5%)		4 (18.2%)		0.27
**Discharge disposition and in-hospital outcomes**							
Ventilatory days	14 (1–115)		13.5 (1–115)		17 (2–51)		0.33
ICU length of stay	23.5 (2–123)		17.5 (2–123)		27.5 (2–62)		0.06
Hospital length of stay	184 (2–1900)		25 (2–137)		39.5 (2–81)		0.16
Follow-up (Days)	184 (2–1900)		152 (2–1900)		228 (9–1810)		0.28
**Disposition**							0.07 for all
Long-term rehabilitation	31 (36.5%)		19 (30.2%)		12 (54.5%)		
Discharge home	19 (22.4%)		17 (27.0%)		2 (9.1%)		
**Mortality**	35 (41.2%)		27 (42.9%)		8 (36.4%)		
30-day mortality	20 (57.2%)		17 (63.0%)		3 (37.5%)		0.39 for all
> 30-day mortality	15 (42.8%)		10 (37%)		5 (62.5%)		
**Cause of death**							0.19 for all
Head injury	20 (57.1%)		17 (63.0%)		3 (37.5%)		
Multiorgan failure	9 (25.7%)		6 (22.2%)		3 (37.5%)		
Septic shock	5 (14.3%)		4 (14.8%)		1 (12.5%)		
Cardiac arrest	1 (2.9%)		0 (0.0%)		1 (12.5%)		
Multiorgan failure	9 (25.7%)		6 (22.2%)		3 (37.5%)		
Septic shock	5 (14.3%)		4 (14.8%)		1 (12.5%)		
Cardiac arrest	1 (2.9%)		0 (0.0%)		1 (12.5%)		

MLI score showed significant difference between the two groups (ECMO 3.35 ± 0.62 and non-ECMO 2.06 ± 0.47; p = 0.001). According to MLI score, ECMO group showed higher rate of severe lung injury (86.4% vs 11.1%, p = 0.001) in comparison to non-ECMO group (Table 1). Surgical interventions were comparable between the 2 groups, however, in ECMO group, there were higher proportion of tracheostomies (63.6% vs 34.9%; p = 0.01) and ICP insertion (54.5% vs 11.1%; p = 0.001) (Table 3).

The rate of MOF was higher in ECMO group (31.8% vs11.1%, p = 0.02) (Table 1). Whereas, the rate of VAP and sepsis were similar between the two groups (p = 0.81 and 0.09; respectively). Hemorrhagic complications were infrequent and comparable such as DIC in 4 patients (2 in each group), thrombocytopenia in 5 patients and GI bleeding in 3 patients. PE and CVA were also comparable in the 2 groups while the number of DVT was higher in the ECMO group (p = 0.02).

Although, it was statistically non-significant, the ECMO group had longer median ventilator days (17 vs. 13.5 days), higher ICU length of stay (27.5 vs.17.5 days) and longer hospital LOS (39.5 vs. 25 days). Almost half of ECMO cases were transferred to long-term rehabilitation whereas, one third of non-ECMO cases did.

### AKI

**Figure 2:**
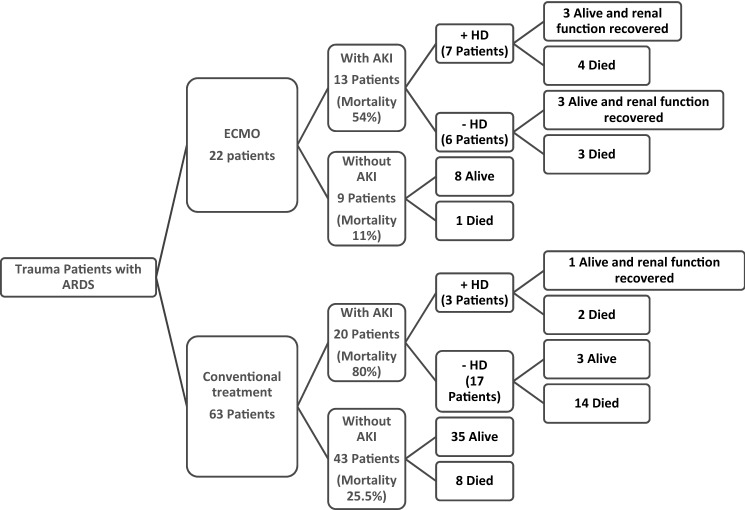
The distribution and outcome of acute kidney injury (AKI) among ADRS patients.

The rate of AKI was higher in ECMO group (59.1% vs 31.7%, p = 0.02) (Table 1). AKI requiring hemofiltration dialysis (D-AKI) was significantly higher in the ECMO group (31.8% vs 4.8%; p = 0.002). Figure 2 shows the distribution and outcome of AKI among ARDS patients. In ECMO group, the mortality was higher among AKI patients in comparison to non-AKI patients (54% vs 11%). Hemodialysis (HD) was required in almost half of ECMO AKI cases, and mortality was almost similar in HD and no HD cases. Renal function was normalized after HD in the survivors. The mortality was higher in AKI non-ECMO (80%) than AKI ECMO group (54%).

### Mortality

**Figure 3:**
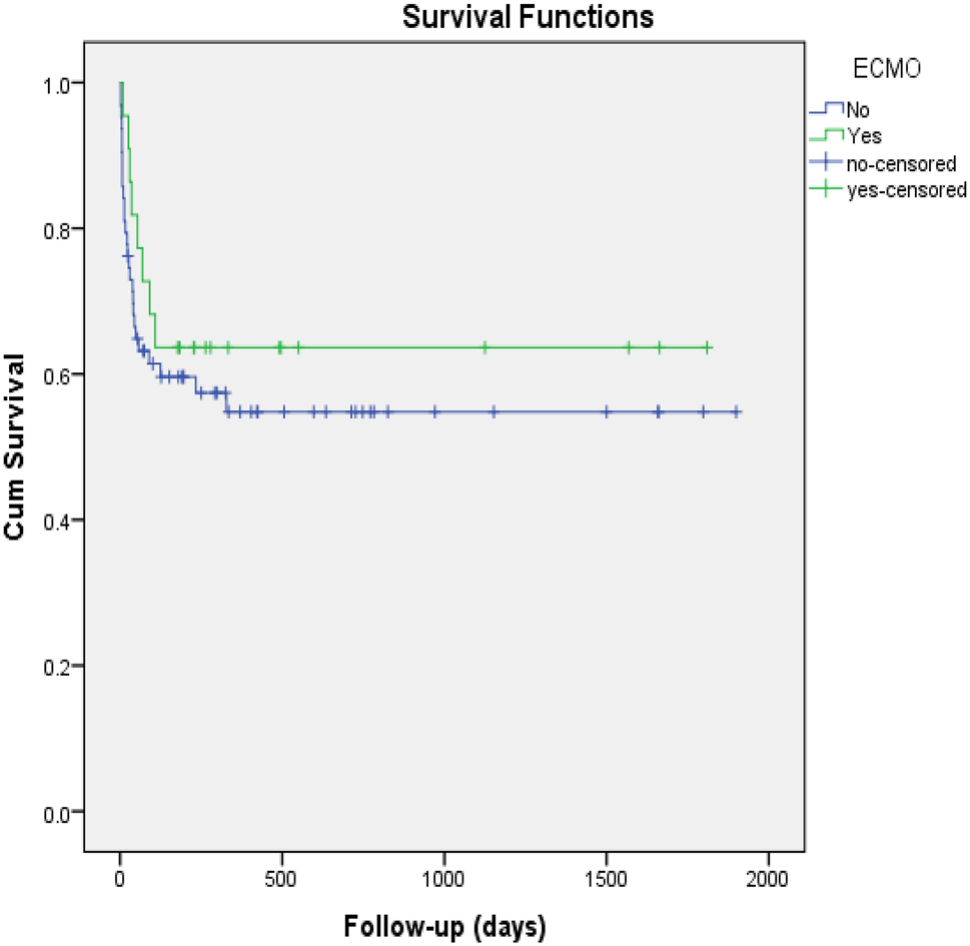
Kaplan–Meier survival curve analysis for ARDS patients with and without ECMO.

The 30-day mortality tends to be higher in the non-ECMO than the ECMO group (63% vs 37.5%) whereas, the pattern of mortality during follow-up period was reversed with a higher rate noticed among ECMO group (62.5% vs 37%). The differences in mortality were not statistically significant (Table 3). The cause of death in the non-ECMO group was mainly TBI (63%) followed by MOF (22.2%) and sepsis (14.8%), whereas in the ECMO group it was 37.5% TBI, 37.5% MOF, 12.5% sepsis and 12.5% cardiac arrest. Kaplan- Meier survival curve analysis (Fig. 3) showed that the survival in the ECMO group was initially close to that of the non-ECMO, however, during the follow-up, the survival rate was better in the ECMO group, but did not reach statistical significance (Log-rank, p = 0.43 and Tarone-Ware, p = 0.37).

Table 4 shows multivariable logistic regression analysis for predictors of mortality among trauma patients with ARDS. AKI (OR 13.03; 95% CI 3.17–53.54, p = 0.001) and brain edema (OR 4.80; 95% CI 1.10–21.03, p = 0.03) were the independent predictors of mortality.

**Table 4:** multivariable logistic regression analysis for predictors of mortality among trauma patients with acute respiratory distress disorder.

Predictor	P value	Odds ratio	95% confidence interval
Age in years	0.573	1.014	0.967–1.063
Injury severity score	0.849	0.996	0.952–1.042
Admission shock index	0.803	1.209	0.272–5.378
Admission GCS	0.516	0.964	0.861–1.078
Sepsis	0.165	2.448	0.692–8.653
Acute kidney injury	0.001	13.034	3.173–53.542
Brain edema	0.037	4.809	1.100–21.028
Extra-axial hematoma	0.717	0.695	0.098–4.956
Murray lung injury score	0.692	1.312	0.342–5.032
ECMO use	0.124	0.158	0.015–1.663

### Subgroup analysis

**Figure 4:**
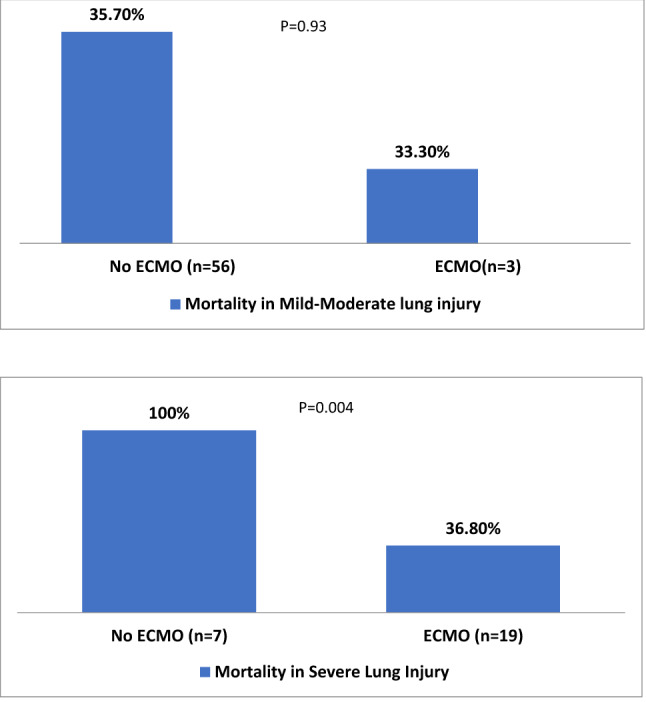
Mortality based on Murray lung injury severity in patients with and without ECMO support.

According to Murray Lung Injury score (severe vs. mild-moderate lung injury), the rate of mortality was compared in Fig. 4. In patients with severe lung injury, non-ECMO use (n = 7) was associated with higher mortality (100% vs 36.8%, p = 0.004) in comparison to the ECMO group (n = 19). However, in mild-moderate lung injury, there was no statistically significant difference in mortality (35.7% vs 33.3%, p = 0.93) between ECMO (n = 3) and non-ECMO group (n = 56).

## Discussion

This is a unique study from a rapidly developing country to evaluate the clinical characteristics, complications, and mortality in young patients with post-trauma ARDS managed with ECMO support versus conventional mechanical ventilation. During the study period, the rate of ARDS in trauma patients was 0.9%, of which one quarter of cases underwent ECMO therapy. Almost, one third of the ECMO group died with severe head injury whereas two thirds of non-ECMO died with head injury. The overall mean SOFA score (9.3 ± 3.4) and on-admission shock index (1.05 ± 0.47) were high which indicates the potential unfavorable outcome. MLI scoring was significantly higher in the ECMO group. The MLI scoring showed that ECMO use was associated with significantly higher survival in patients with severe lung injury than non-ECMO severe MLI group.

The selection of candidates for ECMO is challenging for intensivists due to heterogeneous patient population and the availability of expert team. Moreover, early identification of risk factors of mortality and analysis of long-term outcomes of survivors are necessary to predict the prognosis^23,25^. In our cohort, the overall rate of mortality was 41% and the presence of brain edema and AKI were found to be the independent predictors of mortality. The 30-day survival rate was relatively better in the ECMO group and the long-term survival was better in the non-ECMO group, however these differences in survival were statistically not significant. Our findings indicate that early ECMO (≤ 2 days) was associated with better survival in comparison to delayed ECMO. The literature suggested an overall survival rate after VV ECMO in trauma patients ranges from 50 to 79%^15^ which agrees with our findings (64%). Also, Guirand et al.^26^ showed that ECMO was independently associated with improved survival as compared to the conventional ventilation matched group (age and ISS), however, acute intracranial hemorrhage patients were excluded from this study. An earlier study by Bosarge et al.^27^ reported significant reduction in mortality among the ECMO group (13.3%) compared to the conventional (64.3%) which were matched for age, ISS, TBI, SOFA and MLI scores. Similarly, an observational study reported significantly higher ICU and hospital survival rates in the traumatic extracorporeal life-support (ECLS) cohort as opposed to the non-traumatic ECLS group^28^. In contrast with our findings the EOLIA trial^29^, showed that patients with severe ARDS had no significant reduction in 60-day mortality from early ECMO, as compared to conventional mechanical ventilation.

In our cohort, the duration of mechanical ventilation, ICU and hospital length of stay tends to be prolonged in the ECMO group. Contrarily, Bosarge et al.^27^ showed shorter mean ventilatory days were (7.5 ± 8.4 days) and patients were transitioned to ECMO in a relatively shorter period. Over 59% of our patients had ECMO started within 48 h of admission. On the contrary, in a study of 7 trauma patients by Strumwasser et al.^30^, the survivors had ECMO initiated later than non-survivors (15 vs 7.8 days).The ELSO registry study^12^, demonstrated the mean duration of extracorporeal life support was 8.8 ± 9.5 days which is slightly higher [median 9.5 (1–29) days] in our cohort.

### Trauma, AKI and ECMO

The incidence of AKI in trauma patients varies from 1 to 50% whereas its incidence in patients treated with ECMO ranges between 26 and 85% based on several factors related to patients profiles (age and comorbidity), AKI definition, the clinical scenario, and type of ECMO cannulation (AKI was higher with VA than VV)^18,31,32^. The mortality of AKI patients while on ECMO is around 62–68%^31,32^. A recent study by Chen et al.^33^ showed a higher rate of all-cause mortality in patients with D-AKI (52.3%) as compared to those without D-AKI (33.3%). Also, the long-term mortality in patients survived > 90 days after hospital discharge was significantly lower in patients without D-AKI (22.0%) than those required long-term dialysis (50.0%).

In our cohort, AKI was reported in 38.8% of cases (n = 33), of them 13 cases had ECMO. AKI was associated with higher mortality in patients with (54%) and without ECMO therapy (80%). The ECMO group with AKI had higher mortality in comparison to those without AKI (11%).

Several studies have shown that the RESP score is a useful tool for prediction and discrimination of survival probabilities in ARDS patients treated with ECMO^23,25,34^. In the present study, over 81% of patients in the ECMO group had risk Class IV-V.

Comparison of the long-term outcomes of ARDS patients treated with ECMO and conventional ventilation strategies showed no significant difference in the 1-year survival, but the non-ECMO group had greater impairment of health-related quality of life^35^. It has been suggested that the long-term functional limitation in ARDS survivors is not related to the degree of pulmonary dysfunction at admission, but rather to the consequences of invasive treatment at ICU and severity of illness^25,36^. In our study, 18% of ECMO and 25% of non-ECMO group were discharged home, while higher proportion of patients from ECMO group (50%) were transferred to long-term facility with variable degree of disability as opposed to non-ECMO group (25.4%). Swol et al.^12^ reported ECLS survivors, 23% were discharged home, 19% were transferred back to the referring hospital, and 58% were discharged to another facility.

In our cohort, VAP, sepsis, gastrointestinal bleeding and thromboembolic events such as PE and DVT were the frequent in-hospital complications. Our findings are consistent with the previous studies reported bleeding, nosocomial infection and thromboembolic events as ECMO-related complications. Bleeding occurs in about 20–40% of patients on ECMO with various degrees of severity^12,27,37,38^. Also, nosocomial infections are common in ECMO patients which ranges from 11.7–64%^39,40^. Occurrence of limb ischemia is an uncommon event in trauma patients^12^ which is also evident from our findings (1.2%). Luyt et al.^41^ reported cerebral bleeding (7.5%) and ischemic stroke (2%) as the neurological events in brain injury patients on VV-ECMO. However, in the present study, overall stroke was reported in 4 patients (4.7%), with only one patient in the ECMO group (4.5%).

In our study, the presence of brain edema and AKI were found to be the independent predictors of mortality. While other studies suggested that ISS > 35 and refractory post-traumatic shock/cardiac arrest were independent predictors of hospital mortality^1^. Parker et al.^5^ found that only the presence of hemorrhagic shock at admission was significantly associated with mortality in patients requiring ECMO, and not the age or TBI. However, ISS and admission shock index were not found to be independently associated with mortality in our study. The number of pre-ECMO organ dysfunctions has been used as an important prognostic factor. In previous studies, SOFA score was used as a surrogate for organ failure and pre-ECMO central nervous system dysfunction was associated with poor outcome in the RESP score^23,42,43^. Cheng et al.^44^, demonstrated that in adult VV-ECMO patients, pre-ECMO SOFA score > 9, ventilatory day > 4 and immunocompromised status were independent predictors of mortality. However, the mean SOFA score was comparable in patients with and without ECMO in our study.

The use of ECMO in patients with TBI remains controversial. Wu et al.^1^, studied TBI patients before ECMO. TBI was significant in 19 patients, and a heparin-free ECMO was provided to most patients. No TBI re-bleeding occurs, and only one in-hospital death was reported. In our study, 68% of ECMO group and 51% of non-ECMO group had TBI; no cerebral bleeding was observed but three patients with TBI died in ECMO group. According to the above-mentioned findings, ECMO with a heparin-free strategy seems to be safe in patients with minor or drained TBI^1^.

### Limitations

This study has certain limitations that need to be addressed. It is a single center study with a retrospective nature and relatively small sample size. However, it is representative of the country as our trauma center is the only tertiary level 1 trauma center in the country that manages moderate to severe trauma cases including ECMO support (free of cost). Of note, the study cohort was younger in age compared with other studies. The study groups were matched for age, ISS, and AIS but not for MLI scoring, however, multivariable analysis and sub-analysis were performed to mitigate selection bias and to assess the impact of MLI on the outcome. ECMO was used in 3 cases with mild-moderate MLI because of difficulty of prone positioning in 1 abdominal injury case and difficulty in optimizing ventilatory setting in 2 cases with significant head injury. In addition, due to lack of standard treatment algorithm, some patients in both groups have received various treatments at the discretion of the attending physician. In the non-ECMO group, only 5 cases had official referral/consultation to the ECMO team which declined the ECMO support because of poor prognosis. Therefore, the reason of not using ECMO in the rest of the control group was not clearly stated in the data. Also, health-related quality of life post-discharge was not addressed.

## Conclusions

Although ARDS is uncommon in trauma patients, it has high mortality. ECMO therapy was used in a quarter of ARDS cases. AKI and brain edema rather than ECMO use were independent predictors of mortality in trauma patients with ARDS. Of note, even with associated TBI, trauma patients generally tolerate ECMO therapy. Moreover, ECMO use was associated with better survival in patients with severe lung injury according to MLI scoring. Further prospective study is needed to define the appropriate selection criteria for ECMO use, treatment algorithms and strategies in order to optimize outcomes in trauma patients who developed ARDS.

## Data Availability

All data were shown in the study analysis and illustrations. Access to data needs approval from the Qatar national trauma registry and medical research center of HMC.

## Abbreviations

ECMO: Extracorporeal membrane oxygenation
ARDS: Acute respiratory distress syndrome
AKI: Acute kidney injury
TBI: Traumatic brain injury
MLI: Murray lung injury
ISS: Injury severity score
RESP score: Respiratory ECMO survival prediction score
